# A look at the incidence and risk factors for dog bites in unincorporated Harris County, Texas, USA

**DOI:** 10.14202/vetworld.2020.419-425

**Published:** 2020-03-05

**Authors:** Bonnie C. Hasoon, Alyssa E. Shipp, Jamal Hasoon

**Affiliations:** 1 Division of Zoonoses, Harris County Veterinary Public Health, Houston, Texas 77076, USA; 2 Department of Epidemiology and Biostatistics, Texas A&M School of Public Health, College Station, Texas 77843, USA; 3 Department of Anesthesia, Critical Care and Pain Medicine, Beth Israel Deaconess Medical Center, Harvard Medical School, Boston, Massachusetts 02215, USA

**Keywords:** companion animal behavior, epidemiology, injury, overpopulation, public health

## Abstract

**Aim:**

This study examined the incidence, demographic predictors, and map patterns of dog bites to humans in unincorporated Harris County, Texas, USA.

**Materials and Methods:**

Dog bites reported to Harris County Veterinary Public Health (HCVPH) between January 1, 2013, and December 31, 2016, were analyzed in this retrospective cohort study. Canine and victim characteristics and bite circumstances were evaluated to establish risk factors for bites. Geographic location was used to produce choropleth maps.

**Results:**

There were 6683 dog bites reported to HCVPH between the years of 2013 and 2016, with stable incidence rates over time. The incidence was highest for both children and older adults. Dogs with the primary breed of Pit Bull had the greatest frequency of bites (25.07%), with the second highest breed being Labrador Retrievers (13.72%). Bites were more common from intact dogs of both genders, especially from intact males. Persons aged 70+ had the greatest incidence of severe injury (14.09/100,000). A strong correlation between dog bite incidences and stray dogs was found after controlling for the human population and income.

**Conclusion:**

Dog bites remain a largely preventable issue, and risk factors identified in this study can help direct preventative efforts to reduce the incidence of dog bites.

## Introduction

Dog bites are a largely preventable public health issue with hidden costs to the communities [1]. They are a large financial burden in the United States due to their incidence, associated health-care costs, and potential for serious outcomes such as severe injury, infection, or even death [1]. Dog bite injuries started receiving greater recognition as a public health issue around the start of the 21^st^ century. However, despite the increased recognition, the incidence of dog bites remains high; CDC reports that there are 4.5 million dog bites each year in the United States, and the AVMA estimates this incidence to be 4.7 million [2,3]. A high number of dogs living in homes, as well as having multi-dog households, increase the possibility of dog bite-related injuries. It is estimated that there are 89.7 million owned dogs in the United States, not including strays [4]. There are studies that characterize bite risk at various localities across the United States; however, there are few verifiable reports for Houston or Harris County, Texas. There is a lack of data regarding stray populations and bite cases in unincorporated Harris County. Further, most estimates of dog bites in Texas/Houston are simply crude estimates and have limited epidemiological value.

There have been several national studies that attempt to characterize the populations at risk for dog bites. A 1994 estimate from Injury Control and Risk Survey predicted children had a 150% higher bite rate and a 300% higher medically attended bite rate [5]. Reported bites to children tend to be both more frequent and severe. A nationally-representative study of data collected from Emergency Departments (ED) across the United States from 2006 to 2008 found that 2.3% of ED visits for dog bites resulted in hospitalization [6]. The Insurance Information Institute reports that dog bite claims totaled up to $600 million in 2016 [7]. Biting dogs that get reported tend to be larger, such as Pit Bulls, German Shepherds, or Rottweilers [8]. This should be expected because big dogs can physically do more damage if they do bite [1]. Smaller dogs have certainly been shown to bite as well but may go underreported (with the exception of Chihuahuas) if they did not result in serious injury [9]. Pit Bulls, in particular, have been found to inflict bites that result in serious trauma or death [8,10]. It is important to consider that the breed of the biting dog may not be accurately recorded, mixed-breed dogs are commonly described as if they were purebreds, and the number of dogs of a particular breed in a community is not known [1]. Testosterone in intact dogs has been shown to increase aggressive behavior by making dogs react more intensely and for a longer period of time [8]. Intact males are also involved in 70-76% of reported dog bite incidents [11]. In addition, many studies have reported that more dog bites occur from owned dogs rather than strays [8]. Neighbor-owned dogs have the highest rate of bites, followed by dogs owned by the victim’s family [12]. Strays are reported as having the lowest bite rate, yet stray bites are more commonly reported than others [12]. This may be due to the perceived risk of disease from strays. Results from population-based surveys suggest that <10% of bites are from stray dogs [12]. However, in the 2010 Health of Houston Survey, 37% of participants reported that stray dogs/cats posed an issue, and strays were the most frequent environmental issue reported – greater than crime, air pollution, dumping, and other problems [13].

This study aimed to summarize descriptive characteristics of victims and biting dogs, as well as to monitor trends of dog bite cases in unincorporated Harris County, Texas, USA 2013-2016. With the limited current analysis of bite case data from Harris County, this study hopes to discover whether findings are consistent with national trends. Increasing our understanding of the overall dog bite situation helps raise awareness of this important public health problem. Future analysis could focus on consolidating data from other major cities across the nation to develop a larger perspective.

## Materials and Methods

### Ethical approval

Ethical approval was not necessary for this study. However, approval to conduct the study was obtained from Harris County Veterinary Public Health, Houston, Texas, USA.

### Case selection criteria

Electronic bite case records from 2013 to 2016 from Harris County Veterinary Public Health (HCVPH), the local rabies control authority for unincorporated Harris County, Texas, USA, were used to identify dog bites and scratches to humans reported during this period. As per Texas law, it is required that all bites in unincorporated Harris County be reported to this agency. Unincorporated Harris County includes portions of Harris County that fall outside of the City of Houston and other smaller municipalities. As of December 31, 2016, the population of unincorporated Harris County was estimated to include over 2 million residents.

The reports contained the date, time, location, and circumstances of the bite. The age of the victim, type of injury, and body part location of bite are also included. The dog’s primary and secondary breed, sex, and rabies vaccination status are documented. The dog’s owner is also listed if available. The database also provided information on the location of where stray dogs were picked up and admitted to HCVPH during 2013-2016. This included strays picked up by the local animal control and citizens in the community.

### Bite case records review

Records were reviewed and data extracted regarding the date of the bite, circumstances of the bite, age of victim, type of injury, body part location of bite, dog ownership status, primary breed, and sex. A binary variable for the victim’s ownership of the dog was generated by comparing the owner’s ID with the victim’s ID (or guardian’s ID if the victim was a minor). If the two matched, the dog was said to be owned by the victim. A binary variable for stray status was generated as well, with any dog without an owner ID considered a stray. Age was categorized to match available data on the population of unincorporated Harris County. Victim age equal to 0 was marked as missing data, as the procedure for entering age into the bite reports resulted in multiple meanings for this value.

From the circumstances reported on the bite case, provoked status, dog fight-related status, and stray fight-related status were ascertained for the year 2016. The definition used to determine provoked status was as follows: “Approaching a dog with the intentions to pet/touch the dog or the dog being picked up, petted, hit, kicked or struck with any object or part of a person’s body or any part of the animal’s body having been pulled, pinched, or squeezed whether intentional or unintentional. Also getting bit when feeding a dog or breaking up a dog fight was labeled as provoked.” Dog fight-related bites were defined as a person getting bit or scratched when two or more owned dogs were fighting, while stray fight-related bites involved a person getting bit or scratched when a stray dog was involved in fighting with an owned dog.

### Statistical analysis

Categorical variables were tabulated, and spike plots were generated for continuous variables. Inaccurate records, such as values of “−1” for victim age, were replaced as missing. Each categorical variable was tabulated to find percentages in each category. Mean and 95% confidence intervals were found for each continuous variable. These steps were repeated for each year.

The incidence of dog bites was calculated for each year according to population estimates for unincorporated Harris County based on US Census Bureau and Harris County Appraisal District data (Table-1). Age-specific incidence rates were calculated using 2010-2015 US Census Bureau data, determined based on the 592 Census block group polygon centroids that fall within unincorporated Harris County.

**Table-1:** Dog bite incidence rates, 2013-2016.

Year	Dog bites	Estimated population	Incidence per 10,000	95% CI for incidence proportion
2013	1543	1,747,000	8.83	(8.39, 9.27)
2014	1633	1,810,000	9.02	(8.58, 9.46)
2015	1735	1,942,000	8.93	(8.51, 9.35)
2016	1772	2,030,000	8.73	(8.32, 9.14)

CE=Confidence interval

A binary variable for severe injury was created to include all injuries listed as “severe” or “mauling.” Univariate logistic regression was performed to predict the probability of severe injury for the variables of victim age, dog sex, ownership status, and primary breed. Variables with p<0.25 were considered for multivariate analysis. A final model was chosen based on the best subsets method.

Choropleth maps of reported bites and stray admissions to HCVPH according to zip code were created using the maptile program for Stata version 14.0 (StataCorp LLC, USA). All statistical analyses were performed using Stata version 14.0.

## Results

### Incidence of dog bites in unincorporated Harris county

There were 6683 dog bites reported to unincorporated Harris County between January 1, 2013, and December 31, 2016. Based on a 10% significance level, there is no statistically significant difference in the incidence of reported bites from year to year; bite rates have remained stable over the past 4 years.

**Figure-1:**
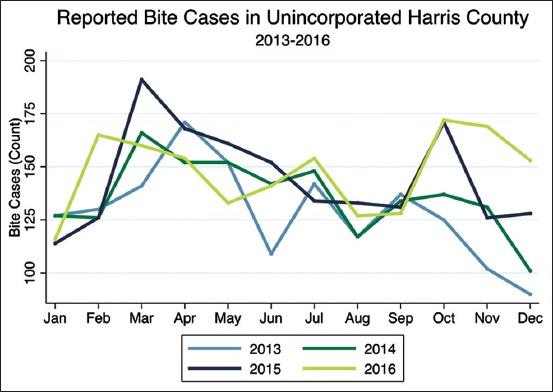
Frequency of reported bites by year, 2013-2016 (n=6683).

Reported bites were similar in frequency for the same time period of each year (Figure-1). On average, the reported number of bites was greatest in March, which all years shared in common, with April and October having the second and third highest frequencies. The years 2015 and 2016 saw an increase in frequency in October. The frequency of bites for each year varied the most in December. There was a progressively larger number of bites occurring in December as the years proceeded.

### Victims and circumstances of dog bites

**Figure-2:**
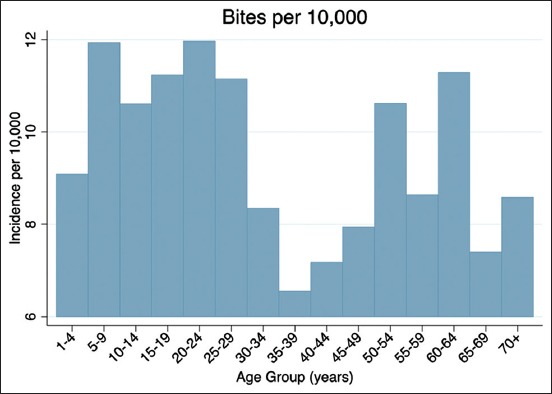
Age-specific incidence rates, 2015.

Of the 6683 reported bites, 20.55% of bites were to children between 1 and 10 years of age. There appears to be a bimodal distribution of bite cases, once as children/youth and once as older adults (Figure-2).

Of all bites, 1665 (24.91%) of the victims were bitten by their own dog. A disproportionate amount of victims bitten by their own dog were middle-aged as compared to the distribution of bites with no regard to ownership. Of victims bitten by their own dog, the most frequently bitten age groups were those aged 40-44 years (11.44%). For perspective, only 6.23% of total bites were for those aged 40-44 years. For victims under 10 years old, only 17.97% of bites were from their own dog, while for victims over 10 years old, 27.31% of bites were from their own dog. Bites by victim-owned dogs did not vary significantly according to year (p<0.129).

Additional analysis of reported bites from 2016 was conducted based on data gathered on additional variables obtained by reading the individual circumstances of each report. In total, there were 1772 reported bites in 2016. Overall, 53.42% of reported bites from 2016 were provoked. Provoked bites are significantly more frequent than unprovoked bites (p<0.01). Intact females have 2.26 times the odds of inflicting an unprovoked bite as compared to spayed females (p<0.001), and intact males have 2.45 times the odds inflicting an unprovoked bite as compared to spayed females (p<0.001). Intact males were 1.78 times more likely to bite unprovoked compared to neutered males (p<0.001).

Overall, 17.77% of reported bites from 2016 involved a dog fight. Of these dog fights, 17.46% involved fights with stray dogs. In total, only 44 cases involved stray dog fights. The greatest frequency of dog fights involved Pit Bulls as the biting dog (32.43%). About 16.22% of dog fights involved Labrador Retrievers as the biting dog. Intact males comprised 37.50% of bites arising from dog fights, greater than any other sex. Overall, intact dogs of either sex inflicted almost double the amount of injuries as fixed dogs as a result of dog fights.

### Biting dog breed, sex, and type of injury

**Figure-3:**
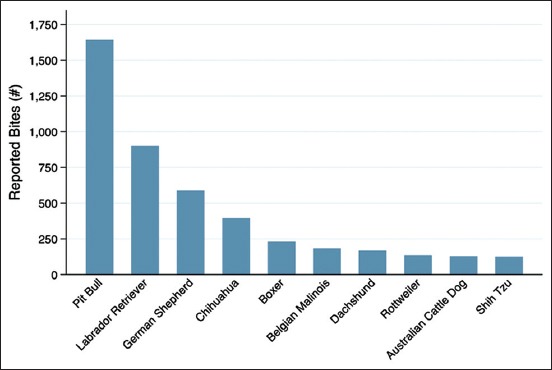
Top ten primary breeds of biting dogs, 2013-2016.

Dogs listed with the primary breed as Pit Bull had the greatest frequency of bites (25.07%), with the second highest breed being Labrador Retrievers (13.72%). The primary breed recorded was reported by the owner of the dog if the dog had an owner, by visual identification by the victim if the dog was never found, or by an animal control officer if the dog was impounded as a stray with no owner coming forward. The percentage of bites from Pit Bulls appears to be consistent through 2013-2016. The percentage of bites from Labrador Retrievers increases each year, beginning at 10.06% in 2013 and increasing at 15.38% in 2016. Labrador Retrievers are the only primary breed that consistently increase each consecutive year. The top ten biting breeds are shown in Figure-3. Dogs listed as intact males accounted for 49.39% of dog bites, followed by intact females (21.8%), neutered males (19.55%), and spayed females (9.26%).

**Figure-4:**
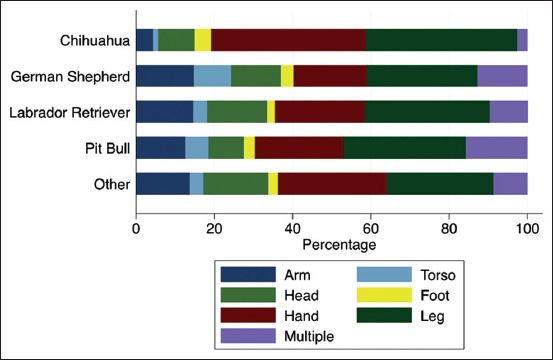
Percentage of bites according to primary breed and body part location.

**Figure-5:**
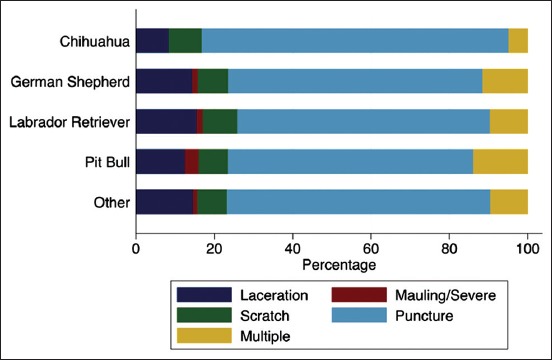
Percentage of bites according to primary breed and type of injury.

Chihuahuas tended to bite more on the hand and leg, and less on the arm (Figure-4). Pit bulls tended to bite multiple body parts more often than other breeds, and German Shepherds bit the torso more often. Chihuahuas had no reported mauling or severe injuries, while Pit Bulls had the greatest percentage of mauling/severe injuries (Figure-5).

### Predictors of severe injury

Severe bites were rare and severe and mauling injuries had a combined frequency of 1.77%. Children under ten had a high incidence of severe injury as compared to other age groups at 8.52/100,000. However, persons aged 70+ had the greatest incidence of severe injury at 14.09/100,000 people. The incidence of severe injury was lowest for those aged 15-19 years at 2.39/100,000 people.

Dog sex, dog breed, and confinement status were all found to be significant predictors of severe injury in the multivariate logistic model. Male dogs are more likely to inflict severe injury, with intact males having the greatest odds. The odds of sustaining a severe injury is twice as likely when the biting dog is an intact male versus an intact female. Pit Bulls had the greatest frequency of severe injuries (49.57%), followed by Labrador Retrievers (12.92%) and German Shepherds (7.69%). The odds of a severe injury by a Pit Bull is 213% higher than the odds for dogs of all other breeds (excluding German Shepherd and Labrador Retriever). The odds of sustaining a severe injury from a dog confined in a household, enclosed yard, or on a leash is 52% greater than for a loose, unconfined dog.

### Correlation between dog bites and stray dogs

In total, 15.55% of bites were from stray dogs. Only 0.97% of bites by strays resulted in severe injury, whereas 1.91% of bites by non-strays resulted in severe injury. With a likelihood-ratio Chi-squared value of 5.2195 (p<0.022), we reject the null hypothesis that severe injury and stray status are independent. Strays have a significantly fewer percentage of severe injury. When looking at the age of victims, strays tend to bite a greater percentage of victims who are between 20 and 55, and a lesser percentage of victims who are younger than 20.

Using census bureau data obtained from the University of Michigan Population Studies Center, the incidence of dog bites was calculated for each zip code. In total, there were 42 zip codes that had twice the incidence of dog bites as compared to the 2016 average for unincorporated Harris County. The top ten highest incidences are shown in Table-2. The highest incidence of 87.96/10,000 (zip code 77562 – Highlands/Barrett) was about 10 times the average incidence of dog bites for unincorporated Harris County in 2016.

**Table-2:** Top ten dog bite incidence rates by ZIP code.

Zip code	Dog bites	Population	Incidence (per 10,000)
77562 (Highlands/Barrett)	92	10,459	87.96
77375 (Tomball/Hufsmith)	258	39,351	65.56
77530 (Channelview/Highlands)	179	31,086	57.58
77389 (Avonack/Willow)	117	21,255	55.05
77532 (Crosby/Barrett)	144	26,236	54.89
77336 (Huffman)	66	12,471	51.92
77447 (Hockley)	61	11,872	51.38
77039 (Aldine)	141	27,562	51.18
77032 (Aldine)	62	12,757	48.60
77373 (Spring)	259	54,609	47.43

**Figure-6:**
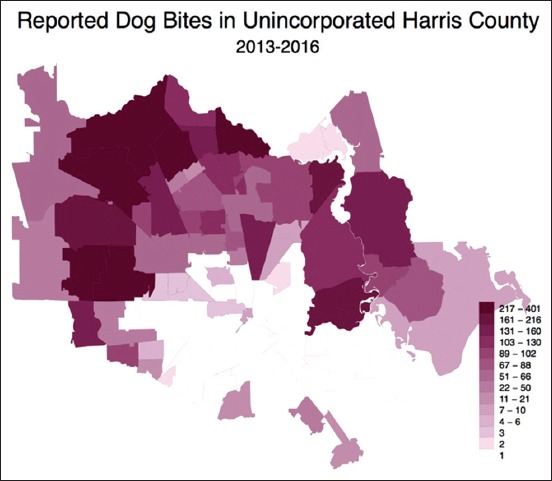
Choropleth map of reported bites by ZIP code.

**Figure-7:**
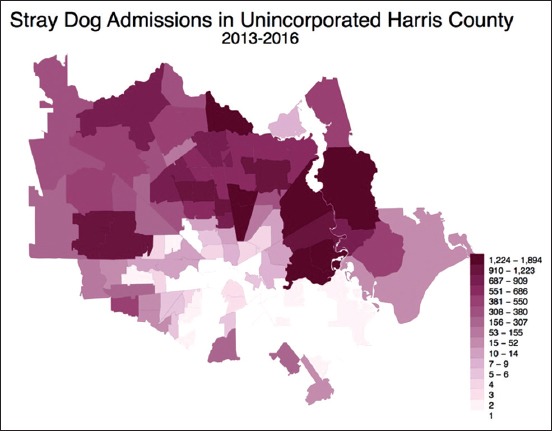
Choropleth map of stray admissions by ZIP code.

**Figure-8:**
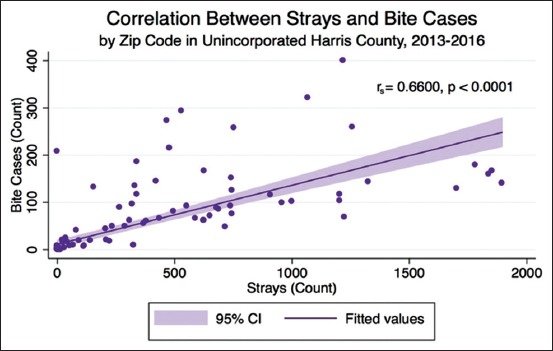
Correlation between stray dogs and dog bites.

Although only 15.55% of bites were from stray dogs, stray dog admissions to HCVPH and reported dog bite locations are significantly correlated (r_s_=0.66, p<0.001) (Figures-6-8). In 2013-2016, 40,577 stray dogs were admitted into HCVPH. Incidence of bites did not appear to correlate with median income (r_s_=0.1522, p<0.1045). Median income was chosen as it is a more robust measure than mean. However, it is important to consider that bites were underreported, and missing data points could potentially strengthen this correlation. The distribution of income was skewed left, which means that we may be missing data for lower incomes. After controlling for both population size and median income, the partial correlation of strays to bites is higher (r=0.7548, p<0.0001). Population size must be controlled since the greater population in a zip code affects both variables. Bites are higher as there are more bodies to be bitten, more pets, and more people present to report strays to animal control. This adjusted correlation suggests that there is a strong correlation between reported bites and stray admissions.

## Discussion

According to Texas laws [14], any person who knows of an animal bite or scratch that could be seen as reasonably capable of transmitting rabies is required to report the incident to the local rabies control authority. In comparison, reported bites in unincorporated Harris County are less than national estimates of bite occurrences. Reported data may underestimate the true incidence of dog bites, as data retrieved from surveys or interviews consistently report higher incidence rates [9]. The 2016 incidence of 8.73/10,000 is only 5.5% of the expected rate based on national estimates [15]. Nonetheless, the lifetime cumulative incidence for dog bites in unincorporated Harris County (based on 2016 incidence) is 6.89%. This means that about 1 in 15 persons would be bitten in their lifetime. Yearly incidence rates appear to be similar to estimates retrieved using similar methods – for example, a study in Oregon using Animal Services data found an incidence of 9.5/10,000 [16]. In spite of gross underreporting, dog bites in unincorporated Harris County are a public health issue to recognize. Future analysis could focus on identifying gaps in reporting through the use of surveys or hospital data.

The frequency of bites according to month is likely to vary according to many factors, including weather patterns and important events. Therefore, it may be more accurate to compare monthly patterns between similar geographic regions. Findings from a study in Los Angeles County found that dog bites peaked in May to August when outdoor activity peaks [17]. While there is no established data from Texas, these findings support our finding that dog bites peak when there are likely surges in outdoor activity.

Similar to the previous findings, our data support the conclusion that children aged 5-9 years are among the most affected. Likewise, it was found that the elderly are more likely to suffer from severe injury. While data on hospitalization were not available, the overall rate of severe bites at 1.77% is similar to the national estimate that 2.2% of bites require hospitalization. The finding that Pit Bulls are more likely to inflict severe injury supports the conclusions of previous studies as well.

The percentage of provoked bites in unincorporated Harris County was higher than the previous estimates [18]. Educating people on recognizing bite warning behaviors could help to prevent people from provoking a bite by interacting with an already distressed or agitated dog. Unprovoked bites are unpredictable – dog behavior training and enforcing leash laws could be two solutions to reduce these attacks, though other solutions may exist. The finding that a greater frequency of bites from intact dogs is unprovoked as compared to fixed dogs could be explained by temperament or territorial behavior in intact dogs. Intact dogs may be more aggressive and tend to bite even when unprovoked. Bites were more common from intact dogs of both genders, but particularly from intact males. Increasing efforts toward educating the public on dog behavior and the benefits of spaying/neutering may prove to be effective at reducing bite rates. Overall, it is important to educate owners to be accountable for and knowledgeable about their pets to reduce the risk of bite-associated injury.

Findings differed slightly in regard to bites from strays. While results from a population-based survey found that <10% of bites are from un-owned dogs, 15.55% of bites in unincorporated Harris County were from strays. However, this may be a result of reporting bias. Victims bitten by strays may be more likely to report the bite due to increased perceived risk of disease. More information detailing the population gap in reporting is needed to reach a decisive conclusion. In comparison to findings from a study in El Paso using similar data collection methods, our data contained half the percentage of victim-owned dogs (20.2% vs. 9.23%) [19].

### Limitations

One limitation lies in the source of data. Data from official reports (rather than survey-based) are likely both underreported and incompletely reported. Because reporting of bites is mandated by law, it is probable that bites may disproportionately come from medical professionals, who are likely to care for more severe bites. It is likely that minor bites and victim-owned bites are underreported, as well. Therefore, this data may have potentially underestimated the true incidence of bites, as well as overestimated the incidence of severe bites.

It is also important to consider that the primary breed of the biting dog may not be accurately reported. The primary breed of mixed-breed dogs is commonly inaccurate [1]. Furthermore, smaller dog breeds may go underreported if they did not result in serious injury [11]. Therefore, although certain breeds may appear to bite in greater frequencies, this is dependent on the distribution of breeds in our population of interest, the accuracy of breed identification, and the factors that motivate an individual to report the bite.

There are unidentified confounders that may mask or alter findings, as well. Characteristics such as socioeconomic status (SES), age, and injury severity may make victims more or less likely to report bites, leading to information bias. It is important to consider characteristics such as population size, SES, and dogs per household when interpreting the results of mapping stray and biting dogs.

Furthermore, there is a portion of data that is missing, likely due to errors in the data recording process. No analysis was able to be done for children <1 year of age, as the value of “0 years” was used in data entry for both infants and unknown age. Due to this issue, 6.88% of the victim age data was reported as missing. However, all other variables used had <5% of data missing (discounting owner ID, where missing value was used to determine stray status).

With regard to the 2016 circumstances data, it is important to acknowledge that the provoked status of 19.19% of reported bites from 2016 could not be inferred due to the situation or description provided by animal control. It is important to consider the reasons for this missing data, as it has the potential to skew our results.

## Conclusion

Increasing our understanding of the magnitude of this dog bite issue helps raise awareness of this important public health problem. In comparison, reported bites in unincorporated HC are less frequent than national estimates. Current data may underestimate the true incidence of dog bites, as data retrieved from surveys or interviews consistently report higher incidence rates. This is likely a reflection of reporting bias and is important to consider when interpreting the results of this study in comparison to established precedents. Considering this limitation, these data are sensitive to identifying bite trends and are easily accessible for local decision-making purposes. Results from this study allow decision-makers to create goals, monitor trends, and design targeted interventions to help reduce local dog bites.

## Authors’ Contributions

BCH conceptualized the aim of the study, designed, planned, supervised the analysis, and corrected the manuscript. AES performed all analyses, prepared the graphs, figures, and tables, and drafted the manuscript. JH provided conceptual support and critically reviewed the manuscript. All authors have read and approved the final manuscript.
